# Evaluation of Biotechnological Active Peptides Secreted by *Saccharomyces cerevisiae* with Potential Skin Benefits

**DOI:** 10.3390/antibiotics13090881

**Published:** 2024-09-13

**Authors:** Elisabete Muchagato Maurício, Patrícia Branco, Ana Luiza Barros Araújo, Catarina Roma-Rodrigues, Katelene Lima, Maria Paula Duarte, Alexandra R. Fernandes, Helena Albergaria

**Affiliations:** 1 BIORG—Bioengineering and Sustainability Research Group, Faculdade de Engenharia, Universidade Lusófona, Av. Campo Grande 376, 1749-024 Lisbon, Portugal; analuiza.araujo.pt@gmail.com; 2 CBIOS—Research Center for Biosciences & Health Technologies, Universidade Lusófona, Campo Grande 376, 1749-024 Lisbon, Portugal; 3 Elisa Câmara, Lda, Dermocosmética, Centro Empresarial de Talaíde, n°7 e 8, 2785-723 Lisbon, Portugal; 4 Linking Landscape, Environment, Agriculture and Food (LEAF), Associated Laboratory TERRA, Instituto Superior de Agronomia, University of Lisbon, Tapada da Ajuda, 1349-017 Lisbon, Portugal; 5 Unit of Bioenergy and Biorefinary, Laboratório Nacional de Energia e Geologia (LNEG), Estrada do Paço do Lumiar, 22, 1649-038 Lisbon, Portugal; helena.albergaria@lneg.pt; 6 UCIBIO—Applied Molecular Biosciences Unit, Department Ciências da Vida, NOVA School of Science and Technology, 2829-516 Caparica, Portugal; catarina.rodrigues@ulusofona.pt; 7 i4HB, Associate Laboratory—Institute for Health and Bioeconomy, Faculdade de Ciências e Tecnologia, Universidade NOVA de Lisboa, 2829-516 Caparica, Portugal; 8 Research Institute for Medicines (iMed.ULisboa), Faculty of Pharmacy, Universidade de Lisboa, 1649-003 Lisbon, Portugal; k.lima@campus.ul.pt; 9 The Mechanical Engineering and Resource Sustainability Center (MEtRICs), Chemistry Department, NOVA School of Science and Technology, Universidade NOVA de Lisboa, 2829-516 Caparica, Portugal; mpcd@fct.unl.pt

**Keywords:** *Saccharomyces cerevisiae*, antimicrobial peptides, biotechnological active peptides, biopreservatives, anti-collagenase, anti-inflammatory, anti-ageing

## Abstract

Biotechnological active peptides are gaining interest in the cosmetics industry due to their antimicrobial, anti-inflammatory, antioxidant, and anti-collagenase (ACE) effects, as well as wound healing properties, making them suitable for cosmetic formulations. The antimicrobial activity of peptides (2–10 kDa) secreted by *Saccharomyces cerevisiae* Ethanol-Red was evaluated against dermal pathogens using broth microdilution and challenge tests. ACE was assessed using a collagenase activity colorimetric assay, antioxidant activity via spectrophotometric monitoring of nitrotetrazolium blue chloride (NBT) reduction, and anti-inflammatory effects by quantifying TNF-α mRNA in lipopolysaccharides (LPS)-exposed dermal fibroblasts. Wound healing assays involved human fibroblasts, endothelial cells, and dermal keratinocytes. The peptides (2–10 kDa) exhibited antimicrobial activity against 10 dermal pathogens, with the Minimum Inhibitory Concentrations (MICs) ranging from 125 µg/mL for *Staphylococcus aureus* to 1000 µg/mL for *Candida albicans* and *Streptococcus pyogenes*. In the challenge test, peptides at their MICs reduced microbial counts significantly, fulfilling ISO 11930:2019 standards, except against *Aspergillus brasiliensis*. The peptides combined with Microcare^Ⓡ^ SB showed synergy, particularly against *C. albicans* and *A. brasilensis*. In vitro, the peptides inhibited collagenase activity by 41.8% and 94.5% at 250 and 1000 µg/mL, respectively, and demonstrated antioxidant capacity. Pre-incubation with peptides decreased TNF-α expression in fibroblasts, indicating anti-inflammatory effects. The peptides do not show to promote or inhibit the angiogenesis of endothelial cells, but are able to attenuate fibrosis, scar formation, and chronic inflammation during the final phases of the wound healing process. The peptides showed antimicrobial, antioxidant, ACE, and anti-inflammatory properties, highlighting their potential as multifunctional bioactive ingredients in skincare, warranting further optimization and exploration in cosmetic applications.

## 1. Introduction

The cosmetic market has witnessed a substantial surge in consumer preference for products derived from natural sources. As a result, the cosmetics industry is proactively seeking biological alternatives to enhance product qualities and replace synthetic chemical components [1,2].

Synthetic preservatives are widely used in the cosmetic industry to extend the shelf life of products and prevent microbial growth. However, their use has raised significant concerns due to potential adverse effects on human health and the environment. One major disadvantage is the risk of skin irritation and allergic reactions. For example, parabens, a common class of synthetic preservatives, have been linked to skin sensitivities and dermatitis (Darbre, 2006) [3]. Additionally, some synthetic preservatives, such as formaldehyde releasers, are known carcinogens and can cause long-term health issues upon prolonged exposure [4].

Many compounds sourced from bacteria, fungi, and algae, such as bio-surfactants, vitamins, antioxidants, pigments, enzymes, and biopeptides, present valuable properties in this regard [5,6,7,8,9,10].

Bioactive peptides have gained considerable attention within the cosmetics industry due to their potential to enhance skin health. Peptides exhibit a wide array of biological functions, encompassing antioxidant, anti-collagenase, anti-aging, anti-inflammatory, and antimicrobial properties [11,12,13,14,15], rendering them excellent candidates for inclusion in cosmetic formulations. 

Natural bioactive peptides with promising cosmetic properties come from different sources, such as plants, bacteria, and yeasts [16]. Yeast peptides have garnered significant interest due to their nutraceutical and functional activities. These peptides, derived from yeast proteins, possess diverse bioactive properties. Several authors demonstrated that yeast peptides exhibit strong superoxide anion scavenging, α-glucosidase, and angiotensin-converting enzyme (ACE) inhibitory activities [17,18], indicating their potential in antioxidative, antidiabetic, and antihypertensive applications. Additionally, Bhattacharyya et al. (2003) [19] explored the functional characteristics of a yeast alcohol dehydrogenase peptide, revealing its chaperone-like activity in preventing protein aggregation, thus highlighting its potential therapeutic applications.

The search for effective and safe antimicrobial agents has led to significant interest in antimicrobial peptides (AMPs) due to their potent activity and low propensity for inducing resistance [20,21]. In cosmetic and dermatological applications, AMPs offer promising benefits for skin health [16].

AMPs, naturally occurring components of the innate immune system, play a crucial role in the defense against pathogens and in wound healing processes. Several AMPs have been identified in human skin cells, providing essential protective functions against microbial contamination [22].

Recent studies have highlighted the beneficial activities of AMPs derived from *Saccharomyces cerevisiae*, a species of yeast commonly used in fermentation processes. These AMPs were first identified in the supernatants of the wine strain *S. cerevisiae* CCMI 885, where they were found to be derived from the glycolytic enzyme glyceraldehyde 3-phosphate dehydrogenase (GAPDH) [13,23]. Afterward, these GAPDH-derived AMPs were found to be secreted by several wine *S. cerevisiae* strains [14]. In subsequent research, peptides ranging from 2–10 kDa were isolated from the supernatant of an industrial starter strain, Ethanol-Red [24]. Those peptides were separated through gel filtration chromatography, and four distinct peaks were detected and tested for their antimicrobial activity. One peak with an estimated molecular weight of 8 kDa, showed antimicrobial activity against various microbial contaminants in alcoholic fermentations, and it was determined by mass spectrometry analyses that the Ethanol-Red strain secreted the same GAPDH-derived AMPs [13,24].

Moreover, the accumulation of these GAPDH-derived AMPs on the surface of *S. cerevisiae* cells was linked to their antagonistic activity against *Lachancea thermotolerans* through a cell-cell contact death-mediated mechanism [25]. This discovery underscores the potential of GAPDH-derived AMPs in broader antimicrobial applications. Furthermore, a peptide derived from GAPDH has been implicated in tissue protection through diverse antifungal strategies and epithelial immunomodulation [26], indicating potential applications in skin care. 

Microbiological contamination in topical formulations is one of the most concerning issues, especially for industry, as it can cause product degradation or, in the case of pathogenic microorganisms, can pose a risk to consumer safety. Thus, chemical preservatives are added to cosmetics and pharmaceutical products to protect them against microbial contamination that can arise due to contamination of raw materials, production water, inadequate hygiene standards during manufacture, or inappropriate use by the consumer [27]. So far, preservatives have been used in the concentrations indicated and studied to protect the product from contamination, protecting the consumers from adverse reactions. However, many questions have been raised about the safety of traditional preservation with synthetic preservatives. Approximately 6% of the population shows contact allergies or sensitization to various preservatives and ingredients in formulations, depending mainly on their concentration [28,29]. In fact, synthetic chemical preservatives are often used without specific tests on the effectiveness of the preservative system, essentially because these tests are expensive and very time-consuming [29]. In this situation, manufacturers choose to avoid the risk of contamination by introducing a greater amount of preservative, usually the maximum amount allowed in European Union regulation (EC) No 1223/2009), Annex V [30], with the consequent increase in the probability of cases of sensitivity or even skin allergies. The growing dissemination of scientific studies indicating some toxic effects associated with the safety of synthetic preservatives has encouraged their replacement by antimicrobials of natural and biotechnological origin, leading the pharmaceutical and cosmetic industries to search for alternative methods of preserving topical products [31,32,33]. In fact, diffusion and microdilution plate methods allow us to characterize the antimicrobial activity associated with the extract or compound under analysis but do not allow us to evaluate its preservative efficacy in relation to a real matrix, such as a cosmetic product.

The effectiveness of the preservative system in a real matrix is usually investigated by performing a challenge test. According to some authors, this method is quite rigorous and realistic and makes it possible to predict the interaction of antimicrobial agents with all the other factors and compounds present in a final formulation. In the challenge test, the product is artificially inoculated with the microorganisms most likely to contaminate cosmetics, such as bacteria, yeasts, and molds, where the ability of antimicrobial agents to remove the most contamination is assessed [34,35]. In this test, a cosmetic product should achieve specific log reductions in microbial counts at various time points and without any increase in microbial growth by day 28 (ISO 11930:2019; Alshehrei, 2024) [36,37]. This method is properly described in the European and American pharmacopeia and, more recently, in ISO 11930:2019 [36], and allows the producers to ensure the microbiological safety of the topical product during its useful life [38].

The exploration of *S. cerevisiae*-derived AMPs could pave the way for novel cosmetic formulations that harness their antimicrobial and healing properties, offering a natural and effective alternative to synthetic preservatives. As consumer demand for healthy and sustainable cosmetic products grows, the incorporation of AMPs could represent a significant advancement in the field.

Thus, taking all this together, in the present study we evaluated the antimicrobial activity of these *S. cerevisiae* AMPs against selected dermal pathogens (i.e., *Staphylococcus aureus*, *Streptococcus mutans*, *Streptococcus mitis*, *Steptococcus pyogens*, *Bacillus cereus*, *Enterococcus faecalis*, *Escherichia coli*, *Pseudomonas aeruginosa*, *Aspergillus brasiliensis*, and *Candida albicans*) and investigated their potential skin benefits such as anti-collagenase effect, anti-inflammatory effect, wound healing effect, and antioxidant effect (e.g., superoxide anion radical scavenging activity), as well as their cytotoxicity on dermal cells.

## 2. Results

### 2.1. Antimicrobial Activity of the S. cerevisiae Peptides (2–10 kDa) against Dermal Pathogens

#### 2.1.1. Determination of Minimum Inhibitory Concentration (MIC)

In order to quantify and confirm the antimicrobial activity, the minimum inhibitory concentration (MIC) was determined for 10 microorganisms that have the potential to cause harm to the health of consumers. The results of the MIC tests for the peptides (2–10 kDa) against various dermal pathogens are presented in Table 1. The MIC values, expressed in µg/mL, were compared with the positive controls, vancomycin, norfloxacin, and ketoconazole, where appropriate, and ethanol (4% (*v*/*v*) (negative control). The analysis of the peptides by high-performance liquid chromatography (HPLC) demonstrated the presence of ethanol (8% (*v*/*v*)). Therefore, the maximum concentration of ethanol in the MIC assay is 4% (*v*/*v*), given that the peptides are combined with the growth medium in a 1:1 ratio, and serial dilutions of them were made in the microplate in accordance with the assay methodology.

The findings (Table 1) showed that all the microorganisms tested grew normally without the peptides and with ethanol as the negative control. Additionally, the microorganisms were also tested with appropriate antibiotics (positive controls) to verify the sensitivity of each strain. The antibiotics used as positive controls had MIC values that met the standards for the different microorganisms as defined in the Performance Standards for Antimicrobial Susceptibility Testing (2021) and Performance Standards for Antifungal Susceptibility Testing of Filamentous Fungi (2020) [39,40].

The peptides (2–10 kDa) demonstrated a variable degree of antimicrobial activity against several dermal pathogens (Table 1). The MIC values highlight the efficacy of the peptides, particularly against *S. aureus* with a MIC of 125 µg/mL. The MIC for *B. cereus*, *E. coli*, *P. aeruginosa*, and *S. epidermidis* was found to be 250 µg/mL, showcasing its broad-spectrum potential. The MIC for Methicillin-resistant *S. aureus* (MRSA), *E. faecalis* and *S. mitis* was 500 µg/mL. However, the peptides were less effective against *S. pyogenes* and *C. albicans*, with an MIC of 1000 µg/mL for both.

#### 2.1.2. Peptides (2–10 kDa) Potential Preservative Effect

##### Physicochemical and Microbiological Analysis

After incorporation of the preservative systems, the formulations were divided into sterile 20 g containers and initial quality control was performed for organoleptic characterization, namely color, general appearance, and odor, as well as physical stability, pH, and viscosity measurements. 

The results showed (Table S1) that after the incorporation of all the preservative systems (t = 0), the formulations remained stable (no physical separation was observed after centrifugation, with an initial pH of 5.2 and a viscosity of 18,000 cps, and there were no changes in color, odor, texture or pH and viscosity, after 28 days of study. Microbiological analysis of the base formulation also showed that there was no contamination, and it was eligible for further challenge testing. 

##### Evaluation of the Antimicrobial Protection of a Cosmetic Product-Preservation Efficacy Test-Challenge Test (ISO 11930:2019)

The challenge test, according to ISO 11930:2019 [36] standards, evaluates the antimicrobial preservation efficacy of cosmetic formulations. This test is critical in ensuring that cosmetic products remain free from microbial contamination, thus safeguarding consumer health. The test involved inoculating body milk formulations with a calibrated inocula of key microorganisms: *E. coli*, *P. aeruginosa*, *S. aureus*, *C. albicans*, and *A. brasiliensis*. These microorganisms were selected due to their prevalence and potential to contaminate cosmetic products, according to European Union regulation (EC) No 1223/2009) [30]. The test monitored microbial counts over 28 days at specified intervals (0, 1, 7, 14, 21, and 28 days) [33].

To test the efficacy of peptides as a cosmetic preservative the formulations were prepared under different conditions, where the peptide (2–10 kDa) were tested alone at MIC concentration (Table 1) or in association with synthetic preservatives at lower concentrations (0.3% (*v*/*v*) Microcare^®^ SB+2–10 kDa). This reduction in the concentration of the classic preservative was carried out for all the microorganisms to check for possible synergistic relationships with the peptide being tested. The positive control (0.6% (*v*/*v*) Microcare^®^ BNA + 1% (*v*/*v*) Microcare SB^®^), was used in the proportions recommended by the suppliers and which are described as classic synthetic preservatives, normally used for effective cosmetic preservation, and the negative control (formulation without any preservative). In addition, two further controls were performed and tested in the formulations: the solvent present in the 2–10 kDa peptide (ethanol control) and a synthetic preservative control at a lower concentration (0.3% (*v*/*v*) Microcare^®^ SB) to validate the results.

**Figure 1:**
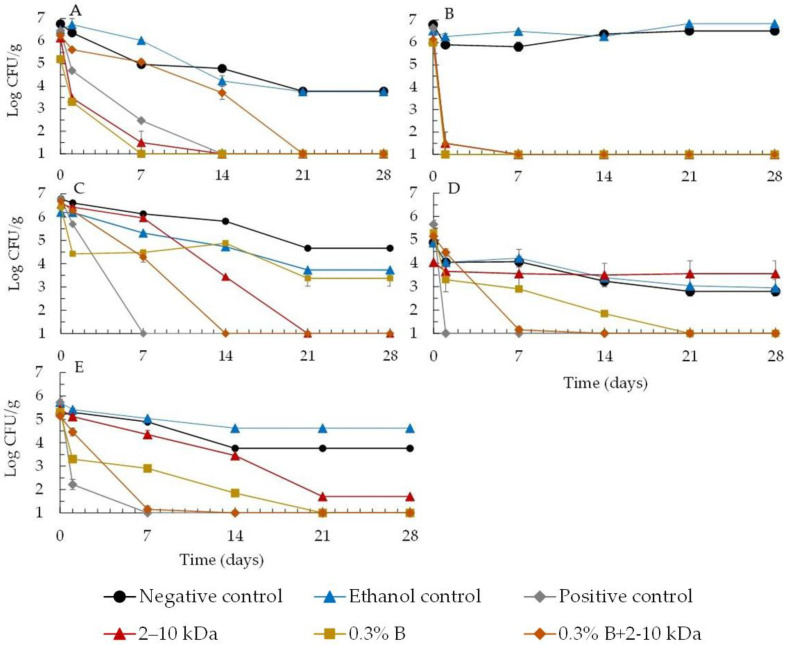
Growth profiles of bacteria, i.e., *E. coli* (**A**), *P. aeruginosa* (**B**), *S. aureus* (**C**) and fungi, i.e., *A. brasiliensis* (**D**) and *C. albicans* (**E**), in body milk formulation in the presence of the peptides (2–10 kDa) at the MIC value and 0.3% (*v*/*v*) of Microcare^®^ BNA (0.3% B), and the association of both (0.3% B+2–10 kDa). A positive control with 0.6% (*v*/*v*) of Microcare^®^ BNA and 1% (*v*/*v*) of Microcare^®^ SB, an ethanol control, and the formulation without any preservative (negative control) were also performed.

In order to analyze the efficacy of the peptides as a cosmetic preservative, a total of 35 formulations were prepared (7 combinations of preservatives × 5 different microorganisms) and the death curves were used to obtain the results shown in Figure 1.

Results showed that for all the assayed microorganisms, the formulation alone without preservatives is not self-preserving and, therefore, the addition of preservatives is required to meet criteria A or B of ISO 11930:2019 [36].

It was observed that the majority of microbial growth in the two negative controls (ethanol control and formulation without any preservative) showed a slight growth reduction in all formulations inoculated with the standard microorganisms. This indicates that the microbial population did not experience substantial mortality due to either the composition of the formulation or the ethanol at the concentration used (4% *v*/*v*). It is normal to observe a decrease in microorganisms during the 28 days of the study, as the base formulation used can cause the microorganisms to die due to the base ingredients; this has also been observed by other authors in similar studies [41,42,43,44]. 

As far as the positive control is concerned, the classical combination of the two synthetic preservatives proved to be effective in the reduction in all the microorganisms tested.

The results showed that peptides (2–10 kDa), when used alone at its MIC, demonstrated significant microbial reduction (Figure 1). However, its efficacy varies among different microorganisms and must be analyzed in order to comply with conservation criteria A and B as defined in the ISO 11930:2019 [36] standards (as shown in the methodology).

In Figure 1A, the peptide (2–10 kDa) was shown to be effective against *E. coli* growth, with a greater reduction in growth at 7 days and 14 days, proven to be more effective than synthetic preservatives (positive control), thus fulfilling criteria A and B of the standard. In this case, the combined addition of a synthetic preservative with peptides (0.3% (*v*/*v*) Microcare^®^ BNA+2–10 kDa) was neither effective nor synergistic, showing an effective reduction only after 14 days.

On the other hand, the peptide (2–10 kDa) proved to be effective in reducing the microbial growth of *P. aeruginosa* (Figure 1B), fulfilling both criteria A and B. In this case, the peptides behaved similarly to the positive control, effectively reducing the microorganism in question. When the peptide was added together with the preservative at a lower concentration (0.3% (*v*/*v*) Microcare^®^ BNA+2–10 kDa), no synergy or advantage was observed in this procedure, with the best results shown for the peptides alone. This result is extremely important, as this bacteria is often associated with the degradation of preservatives and their resistance in cosmetics, which has already been described by several authors. In fact, this microorganism is considered to be one of the most difficult to eliminate from cosmetics, and its resistance to numerous preservation systems is well documented. This high resistance may result from the possibility of this microorganism being able to breakdown some preservatives and surfactants, using them as carbon sources for its development [34,41,42,43].

In Figure 1C, for *S. aureus* the peptides (2–10 kDa) showed a 2-log reduction after 14 days, meeting criterion B. Also, in association with synthetic preservatives at lower concentrations (0.3% (*v*/*v*) Microcare^®^ BNA+2–10 kDa), no synergy or advantage was observed in this procedure, with the best results showing a reduction in the growth of 2 log after 7 days and more than 3 log after 14 days, meeting criterion B of the standard with no increase in growth during the test. So, it can be seen that in this case, the additional addition of 0.3% (*v*/*v*) Microcare^®^ BNA to peptides (2–10 kDa) did not show any advantage or synergy and continued to meet criterion B of the standard. As observed in Figure 1D, the peptides (2–10 kDa) did not show an effective reduction in growth of *A. brasilensis*, with similar values to those observed for the two controls (negative control and ethanol control), thus failing to fulfill criteria A and B of the standard. On the other hand, the peptides, when combined with the synthetic preservative at a lower concentration (0.3% (*v*/*v*) Microcare^®^ BNA+2–10 kDa), showed synergy, thus intensifying the action of the preservative alone and making it possible to observe compliance with criteria A and B of the standard.

As observed in Figure 1E the peptides (2–10 kDa) showed an effective reduction in *C. albicans* growth of more than 1 log after 7 days and 3 log after 14 days, meeting criteria A and B of the standard. In addition, after 7 days of testing, a synergy was observed when the peptide was mixed with a synthetic preservative (0.3% (*v*/*v*) Microcare^®^ BNA+2–10 kDa) reinforcing the effect of the isolated peptide.

It is therefore concluded that the peptides are effective against the tested bacteria and *C. albicans*, showing that they meet the safety criteria required by legislation A or B. It can be seen that the peptides are able to act alone, meeting the A criteria, against, *P. aeruginosa*, *E. coli*, and *C. albicans* and criteria B against *S. aureus* but not against mold *A. brasilensis*. However, when the peptides (2–10 kDa) are used in association with a preservative at a lower concentration (0.3% (*v*/*v*) Microcare^®^ BNA+2–10-kDa), synergy can be observed in both *C. albicans* and *A. brasiliensis*. Therefore, effective preservation of the formulation can be achieved by adding the peptides (2–10 kDa) at MIC concentration to a synthetic preservative in order to comply with the criteria of ISO 11930:2019 [36]. It was also observed that the introduction of the different preservation systems with the peptides 2–10 kDa did not affect the physicochemical and physical stability of the emulsions produced and that at the end of the test, 28 days later, there were no changes in the initial conditions established; they were stable and showed no changes in color, odor, pH, or viscosity.

### 2.2. Potential Skin Benefits of the Peptides (2–10 kDa)

#### In Vitro Collagenase Inhibition Assay

**Figure 2:**
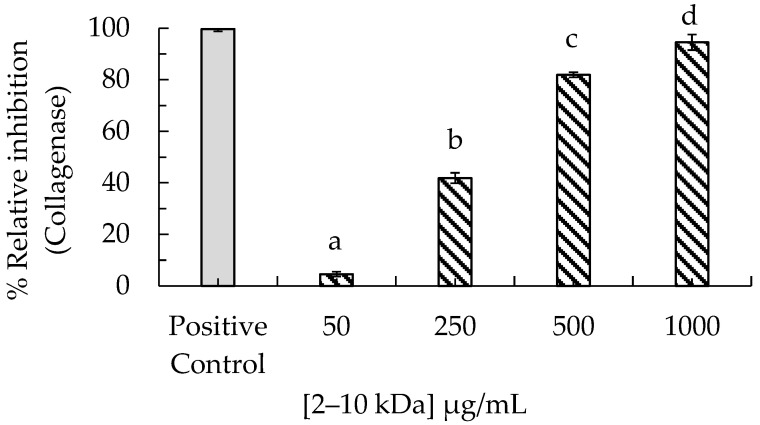
Inhibition of in vitro collagenase activity by 1,10-phenanthroline (positive control) and by peptides (2–10 kDa) at final concentrations of 50, 250, 500, and 1000 µg/mL. Data are presented as means ± SD (error bars) from three independent measurements. Different letters (a–d) indicate significant differences (*p* < 0.05) between the peptide concentrations tested (50–1000 µg/mL).

The peptides (2–10 kDa) were evaluated for their ability to inhibit collagenase. The peptides when tested at a concentration of 250 µg/mL, a reduction of 41.8% in collagenase activity, was observed. Furthermore, the peptides (2–10 kDa) exhibited significant anti-collagenase effects at higher concentrations, with inhibition rates of 81.9% at 500 µg/mL and 94.5% at 1000 µg/mL (Figure 2).

### 2.3. The Peptides (2–10 kDa) Cytotoxicity in Dermal Cells

The cytotoxicity of the peptides (2–10 kDa) in cells from the skin was evaluated on a melanoma cell line, MNT-1, and in normal dermal fibroblasts, keratinocytes, and melanocytes. Cells were exposed for 48h with crescent concentrations of the peptides and their viability was examined using the 3-(4,5-dimethylthiazol-2-yl)-5-(3-carboxymethoxyphenyl)-2-(4-sulfophenyl)-2H tetrazolium, inner salt (MTS) colorimetric assay.

**Figure 3:**
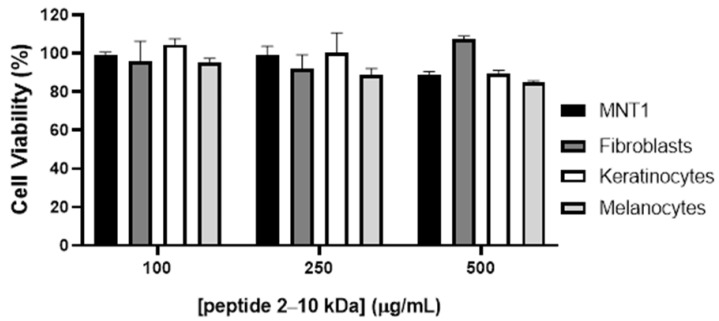
Cytotoxicity of the peptides (2–10 kDa) in melanoma cell line MNT1 and normal dermal fibroblasts, keratinocytes, and melanocytes. Cells were exposed to increasing concentrations of the peptide for 48 h and viability was evaluated with the MTS assay. Bars represent the average ± standard deviation.

Results showed that the peptides (2–10 kDa) present no significant alteration in cell viability until at least 500 µg/mL (Figure 3).

### 2.4. Analysis of Anti-Inflammatory Effect of Peptide (2–10 kDa)

**Figure 4:**
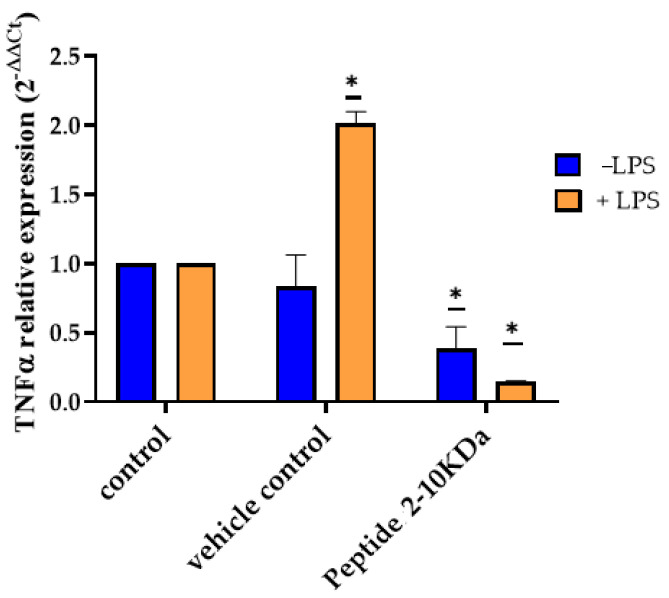
Expression levels of TNF-α in normal dermal fibroblasts after incubation with peptides (2–10 kDa). TNF-α expression after 2 h incubation of fibroblasts with 250 µg/mL of the peptides (2–10 kDa), 1% (*v*/*v*) ethanol (vehicle) or medium (control), followed by further 2 h incubation with (+LPS, orange bars) or without (−LPS, blue bars). Bars represent the average and standard deviation of at least three experiments. * *p*-value < 0.05.

To evaluate the effect of the peptides (2–10 kDa) on the dermal inflammatory process, normal dermal fibroblasts were first incubated with 250 µg/mL of the peptides, with 1% (*v*/*v*) ethanol (vehicle control) or with the same volume of medium (untreated control). After 2 h, they were also exposed to lipopolysaccharides (LPS) and incubated for another 2 h. The level of inflammation was measured by quantification of tumor necrosis factor-alpha (TNF-α) mRNA via RT-qPCR, through comparison with respective untreated control cells. Results showed that incubation with LPS after 2 h exposure to vehicle control resulted in an increased expression of TNF-α (Figure 4). On the other hand, pre-incubation with the peptides resulted in a protective effect against inflammation, with a decrease of TNF-α of 0.15 ± 0.02 in cells incubated first with the peptides and then with LPS to untreated cells incubated with LPS (Figure 4, orange bars), while it was only observed a decrease of 0.39 ± 0.27 in peptides (2–10 kDa) treated cells compared to untreated cells (Figure 4, blue bars).

### 2.5. Analysis of the Effect of Peptides (2–10 kDa) on Angiogenesis

**Figure 5:**
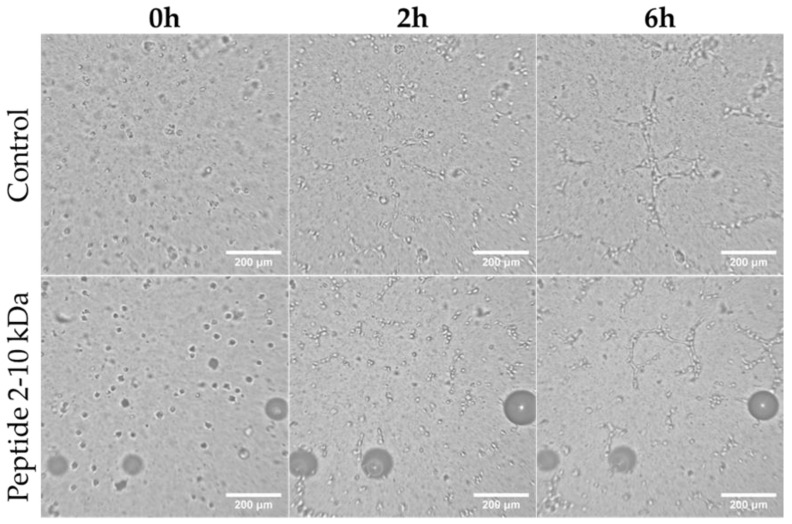
Capillary-like tube formation by HUVEC. Representative images of cells after 0 h, 2 h and 6 h of cells seeding on top of Matrigel in F12-K medium supplemented with 250 µg/mL peptides (2–10 kDa), or 1% (*v*/*v*) ethanol (Control). Scale bar corresponds to 200 µm.

To further understand the effect of the peptides (2–10 kDa) in the proliferative phase of the wound healing process, it was examined the in vitro development of capillary-like tubes by endothelial cells [45]. After HUVEC seeding on top of a matrigel, images were acquired every 15 min, originating a video that allows to evaluate the effect of 250 µg/mL of the peptides (Movie S1 (Supplementary Materials) and representative images in Figure 5) in comparison to the effect of 1% (*v*/*v*) ethanol (vector control, Movie S2 (Supplementary Materials) and representative images in Figure 5). Movie S1 (Supplementary Materials) shows that the formation of tubes in the presence of the peptides starts at 1 h 30 min after seeding and increases until maximum after 6 h (representative images in Figure 5). A similar behavior was observed when cells were exposed to the vector control (Movie S2 (Supplementary Materials), representative images in Figure 5).

### 2.6. Scratch Test

**Figure 6:**
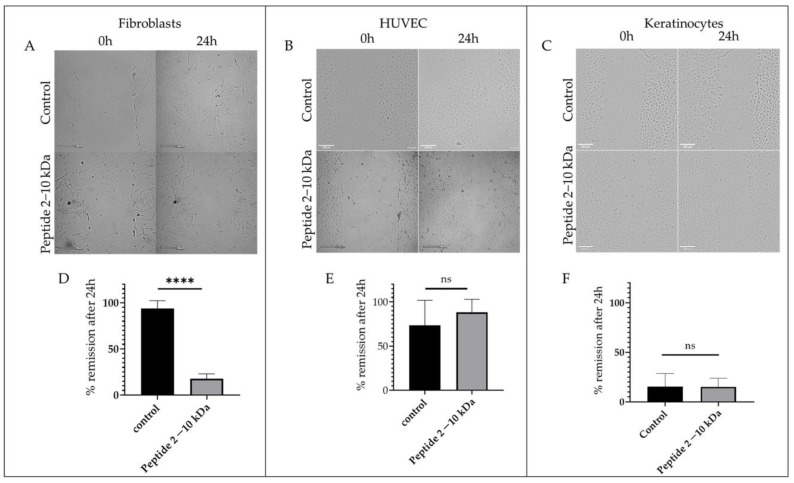
Wound healing assay. Representative images of the wound healing assay at 0 h and 24 h after incubation with 1% (*v*/*v*) ethanol (control) or 250 µg/mL of peptides (2–10 kDa) in (**A**) normal dermal fibroblasts, (**B**) HUVEC, and (**C**) keratinocytes. Scale bars correspond to 200 µm. Percentage of wound scratch closure (% remission) after 24 h incubation with 1% (*v*/*v*) ethanol (control) or 250 µg/mL of peptides (2–10 kDa) in (**D**) normal dermal fibroblasts, (**E**) HUVEC and (**F**) keratinocytes. Bars represent the mean ± SEM of at least two independent experiments. **** *p* value < 0.0001, ns—statistically not significant.

To further understand the peptides (2–10 kDa) effect on the cell migration ability that occurs during the proliferative phase of the wound healing process, a scratch assay was performed in fibroblasts, keratinocytes, and endothelial cells (HUVEC) [46,47]. Results showed that after 24 h, the percentage of regeneration of the scratch in fibroblasts was 93.7 ± 3.0% in the control sample, while it registered a remission of only 17.7 ± 2.7% when cells were incubated with 250 µg/mL of the peptides (Figure 6A,D), suggesting a delay in the migration of fibroblasts in the presence of the peptides (2–10 kDa). On the other hand, there were no significant differences observed in the regeneration of the scratch in HUVEC (Figure 6B,E) and keratinocytes (Figure 6C,F) in the presence of the peptides (2–10 kDa) or in the presence of the vector control.

### 2.7. Superoxide Anion Radical Scavenging Capacity of the Peptides (2–10 kDa)

**Figure 7:**
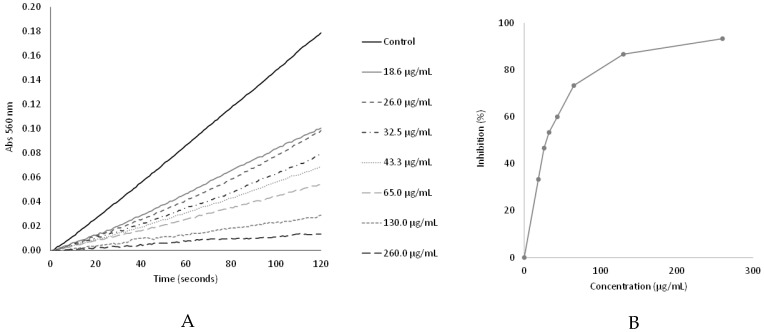
Superoxide scavenging capacity: (**A**) Plot of absorbance (Abs 560 nm) as a function of time for the different peptides concentrations; (**B**) Effect of the peptides on the inhibition of the NBT reduction by the PMS/NADH generated superoxide radical.

The superoxide scavenging capacity was assayed by the inhibition of the nitrotetrazolium blue chloride (NBT) reduction. The antioxidant capacity of the peptides was indicated by a dose-dependent decrease in absorbance at 560 nm, which results from the inhibition of the NBT reduction by the PMS/NADH-generated superoxide radical (Figure 7).

The peptides (2–10 kDa) under study presented a superoxide radical scavenging capacity (IC50 value of 30.6 ± 1.4 µg/mL) close to that of the positive control, gallic acid, (IC50 value of 20.7 ± 3.7 µg/mL) (Table 2).

## 3. Discussion

Both traditional medicine and contemporary scientific research recognize the utility of bioactive peptides in the development of skincare products [48]. This investigation represents the first assessment of peptides secreted by the industrial *S. cerevisiae* strain Ethanol-Red for various bioactivities relevant to skincare, including antimicrobial activity against dermal pathogens, efficacy as a cosmetic preservative (both alone and in combination with synthetic preservatives), collagenase inhibition, antioxidant activity, anti-inflammatory effects, and wound healing properties.

The tested peptides exhibited variable antimicrobial activity against several dermal pathogens (Table 1). In fact, according to the same authors, the antimicrobial activity of natural substances can be defined as strong inhibitors (MIC up to 500 μg/mL), moderate inhibitors (MIC between 600 and 1500 μg/mL), and weak inhibitors (MIC above 1600 μg/mL) [49,50,51]. Using this classification, the peptides demonstrated significant efficacy against both Gram-positive and Gram-negative bacteria, with strong inhibition against *S. aureus* (MIC 125 µg/mL); *S. epidermidis*, *E. coli*, and *P. aeruginosa* (MIC 250 µg/mL); Methicillin-resistant *S. aureus* (MRSA), *E. faecalis*, *S. mitis* (MIC 500 µg/mL), while the peptides showed moderate activity against *S. pyogenes* and *C. albicans*, with a MIC of 1000 µg/mL.

These findings underscore the potential of the peptides secreted by *S. cerevisiae* as natural antimicrobial agents and a potential active ingredient to be incorporated in topical products.

The differential efficacy observed across various pathogens suggests that while the peptide exhibits broad-spectrum activity, its application may be more suitable to inhibit specific microorganisms. Overall, this study highlights the promising role of *S. cerevisiae*-derived peptides in developing effective and natural skincare solutions.

The challenge test results (Figure 1) underscore the importance of selecting appropriate preservatives for cosmetic formulations. The results obtained showed that the peptide can also be used in the preservation of cosmetic formulations. It can be seen that the peptide is able to act alone against *P. aeruginosa*, *E. coli*, *C. albicans*, and *S. aureus* complying with the criteria A and B of the f ISO 11930:2019 [36] but not against mold *A. brasiliensis*. However, when the peptides (2–10 kDa) were added in association with a preservative at a lower concentration (0.3% (*v*/*v*) Microcare^®^ BNA+2–10 kDa), synergy can be observed in both *C. albicans* and *A. brasiliensis* (Figure 1). The co-preservation system (0.3% (*v*/*v*) Microcare^®^ BNA+2–10 kDa) appears to be the most suitable for preserving the emulsion studied, allowing the concentration of the conventional preservative to be reduced. This result may be mainly due to the possibility of having established some synergistic relationships between the peptides and the Microcare^®^ BNA preservative, which proved to be efficient in accordance with the efficacy criteria A and B. Similar results were also described by Kunicka-Styczyńska, et al. (2011) [52] when they compared the antimicrobial efficacy of a conventional synthetic preservation system with a plant-based preservation system, these authors also found a synergistic effect between the two systems. In fact, the use of natural substances as potential preservatives in cosmetics has been mentioned by some authors [33,51]. However, they have found that it is often only when combined with other preservatives or substances with antimicrobial properties that they can ensure the strongest and longest-lasting preservation of cosmetics [32,53]. Thus, based on the results of this study, the possibility of using the peptides in combination with a reduced preservative system for the correct preservation of the emulsions makes it possible to reduce the dose of conventional preservatives recommended by the suppliers. This also makes it possible to reduce the toxic effects often associated with the use of these chemical synthetic preservatives.

It should also be emphasized that any change to the formulation will necessarily require a new challenge test to be performed due to the complexity and diversity of topical formulations and the possibility of interaction between the different ingredients of the formulation.

The *S. cerevisiae* peptides (2–10 kDa), particularly when combined with Microcare^®^ BNA, demonstrate promising preservation efficacy, potentially allowing for reduced concentrations of synthetic preservatives. These findings highlight a pathway towards safer, more effective cosmetic products with optimized preservative systems, which align with the growing consumer demand for natural and less harmful ingredients in skincare formulations.

Collagen is the most abundant protein in the extracellular matrix (ECM) of skin and plays a crucial role in maintaining tissue structure and function [54,55]. However, excessive collagen degradation by collagenases can lead to tissue damage and contribute to the progression of several diseases, including arthritis, chronic wounds, and skin aging [55,56,57]. Therefore, the search for natural inhibitors of collagenases has gained significant attention. Natural agents with anti-aging properties, such as plant extracts and natural peptides from fruits, spirulina, and yeasts, have been reported [58,59,60,61,62]. Peptides and proteins exhibit notable anti-collagenase activities, which are of significant interest for various therapeutic and cosmetic applications. For instance, Ennaas et al. (2016) [63] identified collagencin, an antimicrobial peptide from fish collagen, which has potential antibacterial properties and interactions with membrane structures, indicating its relevance in skin health and food safety. Also, Ryu et al. (2010) [64] isolated a novel peptide (SPH-1) from a seahorse, that inhibits of the NF-κB/p38 signaling pathway, blocking the phosphorylation and activation of NF-κB and p38 kinase, which are critical signaling molecules in the expression of collagenases 1 and 3 [64]. By inhibiting this pathway, this peptide reduces the production of these collagenases, thus preventing collagen degradation.

The present study found that the peptides secreted by *S. cerevisiae* were able to inhibit collagenase activity in a dose-dependent manner, with inhibition percentages of 41.8% and 81.9% for concentrations of 250 and 500 µg/mL, respectively (Figure 2), highlighting their potential as a natural inhibitor of collagenase and their applicability in anti-aging skincare products.

Wound healing is a complex process triggered after tissue injury, involving a complex network of different types of cells, and molecular mediators including cytokines and the endothelial system [47,65]. The process is divided into four major steps: (i) hemostasis, which occur immediately after tissue damage, and consists of blood vessel constriction, formation of a platelet plug, and fibrin production to seal the wound and prevent excessive bleeding; (ii) inflammatory phase, occurs after 2–3 days, and consist in the attraction of neutrophiles and macrophages to remove debris and bacteria; (iii) the proliferative phase, which happens 2 to 10 days after the tissue injury, and is associated with fibroblasts migration with consequent production of collagen and other extracellular matrix components, and release of cytokines that triggers angiogenesis and epithelialization with migration and proliferation of epithelial cells to cover the wound surface; and lastly, the remodeling phase, which begins 2 to 3 weeks after the tissue damage, and is associated with the rearrangement and realignment of the collagen fibbers in the wound to enhance tissue strength and scar formation [47,65]. The four stages are tightly regulated through growth factors, cytokines, and other signaling molecules, resulting in a dynamic process [47,66]. Depending on the physiological condition of the organism, wound healing may result in wounds that heal with minimal scars, fibrosis, or in chronic wounds [47,67]. Fibrosis is a process that may arise during the remodeling phase and result in hypertrophic scars and keloids, which have detrimental effects since it impairs tissue function and has an aesthetic impact on the patient [67]. It is characterized by aberrant extracellular matrix deposition, fibroblast accumulation, and chronic inflammation [67]. Chronic wounds are generally characterized by a nonmigratory and hyperproliferative epidermis, the presence of infection with biofilm formation and unresolved inflammation, exhibiting elevated levels of pro-inflammatory cytokines, like TNF-a [66]. Taken together, results showed that fibroblast incubation with the peptides under study resulted in a decreased cell migration (Figure 6) and a protective effect against inflammation induction (Figure 4). Moreover, incubation of HUVEC with 250 µg/mL of the peptides (2–10 kDa) showed no significant alterations in the endothelial cells’ movement (Figure 6) or angiogenic ability (Figure 5). The keratinocyte’s movement was also not affected by the presence of the peptides (Figure 6F). Although the reduced fibroblast migration in the presence of the peptides makes it less suitable for its application during the initial proliferative phase of the wound healing process, its anti-inflammatory activity, together with antibacterial and antioxidant activities, and no cytotoxicity at concentrations up to 500 µg/mL (Figure 3), makes the peptides suitable for application during the late proliferative phase or in the beginning of the remodeling phase of the wound healing process. In fact, by attenuating fibroblast migration, the application of the peptides may reduce fibrosis, and consequently minimize the aesthetic impact of the scar and loss of tissue characteristics and counteract the mechanisms that are impaired in chronic wounds, including chronic inflammation or bacterial growth [65,66].

The superoxide anion radical can arise when a single electron is supplied to molecular oxygen. The most important source of superoxide radical in vivo is probably the mitochondrial electron-transport chain [68]. However, this radical can arise also from other enzymatic and non-enzymatic reactions that use molecular oxygen as an electron acceptor [69]. The oxidizing power of superoxide itself is moderate, but other radicals and reactive intermediates, like hydrogen peroxide, hydroxyl radical, or peroxynitrite radicals, are formed in reaction sequences starting with superoxide anion radical [68,70]. Thus, the production of superoxide can give rise to more powerful and reactive species and eventually trigger the state of oxidative stress, with the occurrence of oxidative damage to important cellular constituents such as DNA, proteins, and lipids. Oxidative stress plays an important role in the development of several diseases and in the aging process, including the skin aging process [70,71].

The peptides secreted by *S. cerevisiae* under study presented a superoxide radical scavenging capacity close to that of the positive control (gallic acid) (Table 2) and higher than those reported to other peptides, namely to peptides derived from chicken skin gelatin hydrolysate [72] or to peptides from collagen hydrolysate of redlip croaker (*Pseudosciaena polyactis*) scales [73]. Factors influencing peptides’ antioxidant capacity include their amino acid composition, sequence, and molecular weight [74]. Hydroxyl groups present in aromatic amino acids, like tryptophan, tyrosine, and phenylalanine, play a critical role in free radical scavenging [72]. Additionally, histidine, proline, valine, leucine, methionine, glycine, and alanine have been reported to contribute to antioxidant capacity [71,72,74], The superoxide radical scavenging capacity of the peptides suggests its potential to reduce oxidative skin damage, thereby potentially slowing the skin aging process.

These findings highlight the potential of the peptides secreted by *S. cerevisiae* under study as natural antimicrobial agents in skincare products. These peptides have been demonstrated to be effective against both Gram-positive and Gram-negative bacteria, and their synergistic effects when combined with Microcare^®^ BNA suggest a viable pathway for reducing the concentration of conventional synthetic preservatives in cosmetic formulations. This approach not only fulfills consumer expectations regarding the use of natural and safer ingredients, as well as prevents skin allergic reactions and avoids synthetic preservatives that might provoke health problems [3,4]. Further research and optimization are required in order to fully harness the capabilities of these peptides, particularly their applicability across diverse cosmetic formulations. These developments indicate a significant advancement in the field of skincare products, facilitating the creation of preservative systems that are both effective and compliant with safety standards. This represents an important step towards integrating innovative preservative solutions into the cosmetics industry.

## 4. Materials and Methods

### 4.1. Production of the Peptides (2–10 kDa)

The peptides (2–10 kDa) of *S. cerevisiae* fermentation supernatants were obtained as described in Branco et al. (2014) [13]. Briefly, an alcoholic fermentation was performed in 2 L shake-flasks containing 1 L of a synthetic medium. The synthetic medium consisted of 110 g/L glucose, 110 g/L fructose, an acid solution containing 6 g/L tartaric acid, 3 g/L malic acid, and 0.5 g/L citric acid; an amino acid (aa) solution composed of 1.7 g/L yeast nitrogen base without aa, 2 g/L casamino acids, 0.2 g/L calcium chloride, 0.8 g/L arginine, 1 g/L proline, 0.1 g/L tryptophan, and 2.5 g/L yeast extract, with a pH of 4.5. All solutions were autoclaved, except for the amino acid solution, which was sterilized by filtration (0.22 µm). The medium was inoculated with *S. cerevisiae* Ethanol-Red strain at an initial cell density of 1.0 × 10^5^ cells/mL and then incubated at 25 °C, without agitation, for 7 days. After 7 days, cells were removed by centrifugation and the supernatant was then sterilized by filtration through 0.45 µm Millipore membrane and then by 0.22 µm Millipore membrane (Burlington, MA, USA). Afterward, this cell-free supernatant was first ultrafiltrated through centrifugal filter units (Vivaspin 15R, Sartorius, Göttingen, Germany) equipped with 10 kDa membranes and then the permeate (<10 kDa) was concentrated (40-fold) in similar centrifugal units equipped with 2 kDa membranes.

### 4.2. High-Performance Liquid Chromatography Analysis

The 2–10 kDa chromatography profile (Figure S1) was obtained by gel filtration chromatography, using a Superdex-Peptide column (10/300 GL, GE Healthcare, London, UK) coupled to a High-Performance Liquid Chromatography (HPLC) system (Merck Hitachi, Darmstadt, Germany) equipped with a UV detector (Merck Hitachi, Darmstadt, Germany). Two hundred microlitres of fraction was eluted with ammonium acetate 0.1 M at a flow rate of 0.7 mL/min.

The ethanol concentration in the 2–10 kDa peptidic fraction was analyzed in duplicate using a High-Performance Liquid Chromatography (HPLC) system from Merck Hitachi (Darmstadt, Germany), which was equipped with a refractive index detector (L-7490, Merck Hitachi, Darmstadt, Germany). Initially, the 2–10 kDa fraction was filtered through 0.22 μm Millipore membranes (Burlington, MA, USA) before being injected into a Sugar-Pak column, 10 µm, 6.5 mm × 300 mm (Waters Hitachi, Milford, CT, USA). The elution was carried out with a degassed aqueous mobile phase containing CaEDTA (50 mg/L) at a temperature of 90 °C, with a flow rate of 0.5 mL/min.

### 4.3. Microorganisms and Preparation of Inoculum Cultures

In this work, the following microorganisms from Culture Collection of Elisa Câmara Laboratory were used: Gram-positive bacteria: *Bacillus cereus* (ATCC^®^ 11778); Methicillin-resistant *Staphylococcus aureus* (MRSA ATCC^®^ 33591), *Staphylococcus aureus* (ATCC^®^ 6538), *Streptococcus mutans* (ATCC^®^ 25175), *Streptococcus mitis* (NCIMB^®^ 13770), Gram-negative bacteria: *Escherichia coli* (ATCC^®^ 8739) and *Pseudomonas aeruginosa* (ATCC^®^ 9027); the mold *Aspergillus brasiliensis* (ATCC^®^ 16404) and yeasts: *Candida albicans* (ATCC^®^ 10231) and *Saccharomyces cerevisiae* Ethanol-red, obtained from the Lesaffre Advanced Fermentations company (Marcq-en-Baroeul, France).

A subculture was prepared to create the ATCC working culture of the microorganisms, using the stock culture on plates with the recommended media for each microorganism, following the suppliers’ guidelines and the Clinical and Laboratory Standards Institute [39]. *S. cerevisiae* Ethanol-red was maintained on YEPD-agar slants (containing 20 g/L glucose, 20 g/L peptone, 10 g/L yeast extract, and 20 g/L agar, pH 6) and stored at 4 °C. *S. cerevisiae* Ethanol-red inoculums were obtained by transferring one YEPD-agar slant of each strain (pre-grown at 30 °C for 48–72 h) into 100 mL of YEPD medium (composed of 10 g/L yeast extract, 20 g/L peptone, and 20 g/L glucose) and incubating the cultures at 30 °C with agitation at 150 rpm for 16 h.

### 4.4. Antimicrobial Activity

The antimicrobial activity of the peptides (2–10 kDa) was assessed against all the microorganisms which were mentioned in point 4.3 and determined using the broth microdilution method, according to Clinical and Laboratory Standards Institute guidelines [39]. All assays were performed in triplicate, and positive controls were used for bacteria (vancomycin and norfloxacin at 1 mg/ ml, Merck KGaA, Darmstadt, Germany) or for yeasts (ketoconazole at 0.1 mg/mL, Merck KGaA, Darmstadt, Germany). The negative controls (ethanol at 8% (*v*/*v*)) were also included in all the assays and all the controls were used in the same conditions and dilutions.

#### Minimum Inhibitory Concentration (MIC)

The MIC was assessed using the broth microdilution method in 96-well microtiter plates, as described by Pereira et al. (2023) [50]. Peptides (2–10 kDa) were added at a final concentration of 2000 µg/mL, with the first row undergoing a 1:2 dilution, therefore the maximal concentration tested was 1000 µg/mL for all microorganisms, lowed by a series of 2-fold dilutions in different broths: Brain Heart Infusion (BHI) broth (Biokar Diagnostics, Allonne, France) for *Streptococcus* strains, Sabouraud Dextrose broth (Biokar Diagnostics, Allonne, France) for yeasts and molds, and Mueller-Hinton broth (Biokar Diagnostics, Allonne, France) for the remaining microorganisms. A standardized saline suspension (NaCl, 0.85% *w*/*v*) was prepared to inoculate the plates, with turbidity adjusted to match the 0.5 McFarland standard (DEN-1, McFarland Densitometer, Biosan, Riga, Latvia), equivalent to 1–2 × 10^8^ CFU/mL for bacteria, and 1–5 × 10^6^ CFU/mL for yeasts and molds. The microplates were incubated at 35 °C for 24 h for all microorganisms, except yeasts, which were incubated at 30 °C for 24 h, and molds, which required 5–7 days of incubation at 25 °C. After incubation, the MIC was identified as the lowest concentration of peptides at which no visible microbial growth was observed.

### 4.5. Peptides (2–10 kDa) Potential Preservative Effect

#### 4.5.1. Tested Product

The cosmetic base formulation tested was a nutritive royal jelly cream^®^ (O/W emulsion) without preservation, provided by Elisa Câmara, Lda. (Lisbon, Portugal) and Ingredients in compliance with the International Nomenclature of Cosmetics Ingredients (INCI) are: Aqua, Caprylic/Capric Trygliceride (Trigliceridos MCT; Quimidroga; Barcelona, Spain); Cetearyl Alcohol, Sodium Cetearyl Sulfate (Lanette N^®^; BASF, Germany); Polyglyceryl-3 Methylglucose Distearate (Tego Care^®^ 450; Evonik nutrition and care GmbH, Essen, Germany); Glycerin (Glycerine USP, PH. Eur.; Interfat-natural oils, Barcelona, Spain); Panax Ginseng extract (Ginseng Extract; Provital, Barcelona, Spain); Royal Jelly (Lipoplastidine^®^ Pappa regalis, Vevy Europe s.p.a., Genova, Italy). The formulations were prepared in the Elisa Câmara laboratory in accordance with good manufacturing practices (ISO 22716:2007) [75].

#### 4.5.2. Physicochemical and Microbiological Analysis

To determine the physicochemical parameters of the royal jelly formulations, pH (ISO 4316:1977) [76] (Metrohm; 692pH/Ion Meter 09187, Herisau, Sweden), viscosity (ISO 2555:2000) [77] (Brookfield (sp5/20rpm); HADV-II+RT609008, Worcester, MA, USA) and physical stability were by centrifuging during 1 h at 5000 rpm (P. Selecta; Centronic 3ZZ102, Barcelone, Spain). Also, microbiological analyses were also carried out, testing for enumeration and detection of mesophilic bacteria (ISO 21149:2017) [78], *S. aureus* (ISO 22718:2015) [79], *E. coli* (ISO 21150:2015) [80], *P. aeruginosa* (ISO 22717:2015) [81], *C. albicans* (ISO 18416:2015) [82] and total yeast and molds count (ISO 16212:2017) [83] to ensure that the formulation was not contaminated according standard methods.

#### 4.5.3. Evaluation of the Antimicrobial Protection of a Cosmetic Product-Preservation Efficacy Test-Challenge Test (ISO 11930:2019)

The evaluation of the preservation of a cosmetic formulation is based on inoculation of the formulation with a calibrated inocula. The microorganisms’ standards recommended and used for this test were *P. aeruginosa*, *S. aureus*, *E. coli*, *C. albicans*, and *A. brasilliensis*, which are considered the most important microorganisms that can easily contaminate cosmetics products [36].

This standard method is employed during the product development phase to prevent microbial growth in cosmetic formulations. It determines the necessary preservative concentration for effective preservation by assessing the reduction in microorganisms over a 28-day period. All culture media, diluents, and inocula were prepared according to the manufacturer’s guidelines and in compliance with ISO 11190:2019 standards [36].

Several cosmetic milk emulsions were prepared using the previously described base formulation (see Section 4.5.1), with different concentrations/combinations of preservatives: 1-peptides (2–10 kDa) at MIC concentration; 2-Microcare^®^ BNA (benzyl alcohol at 0.3% (*v*/*v*); 3-Microcare^®^ BNA (benzyl alcohol (0.3% (*v*/*v*)) + peptides (2–10-kDa) at MIC concentration; 4-Microcare^®^ BNA (benzyl alcohol 0.6% (*v*/*v*)) + Microcare SB^®^ (sodium benzoate, potassium sorbate ((1% (*v*/*v*)); 5-Ethanol at 8% (*v*/*v*); 6-formulation without preservative (negative control). Then, the different formulations prepared (samples of 20 g) were introduced in sterile containers, separately and were inoculated with each one of the five standard strains considered to obtain 1 × 10^6^ CFU/g for bacteria and 1 × 10^5^ CFU/g for fungus. All the inoculated formulations were stored and observed for 28 days at 25 °C. Colony counts were performed at contact times of 0, 1, 7, 14, 21, and 28 days, using the plate count method of ISO 11930:2019 [36]. The results were expressed as log CFU/g. All determinations were performed in triplicate.

Thus, the efficacy of the peptides (2–10 kDa) as a cosmetic preservative was further assessed alone at MIC concentration (Table 1) or in association with synthetic preservatives at a lower concentration (0.3% (*v*/*v*) Microcare^®^ SB+2–10-kDa). This reduction in the concentration of the classic preservative was carried out to check for possible synergistic relationships with the peptides being tested. The positive control (0.6% (*v*/*v*) Microcare^®^ SB+ and 1% (*v*/*v*) Microcare^®^ SB was used in the proportions recommended by the suppliers, which are described as classic synthetic preservatives, normally used for effective cosmetic preservation, and the negative control (formulation without any preservative). Additionally, two more controls were performed and tested in formulations: the solvent present in the peptides (2–10-kDa) (ethanol control) and a control for synthetic preservative at a lower concentration (0.3% (*v*/*v*) Microcare^®^ SB) to validate the results.

The formulation’s preservation is deemed effective if it meets either Criterion A or B:

Criterion A-For bacteria, the formulation is effective if the total bacterial count decreases by two to three logarithmic units between the second and seventh days of the challenge test, with no subsequent increase in population until the test concludes on the 28th day. For fungi, the criterion is met if the fungal count is reduced by two logarithmic units by the 14th day, with no further growth until the end of the test. If these conditions are satisfied for all recommended microorganisms, the cosmetic product is considered protected against microorganisms that could pose a health risk to consumers.

Criterion B-For bacteria, the formulation is effective if the bacterial count decreases by three logarithmic units by the 14th day, with no growth observed until the 28th day. For fungi, the criterion is satisfied if the fungal count is reduced by one logarithmic unit by the 14th day, with no growth observed until the test concludes. While meeting this criterion indicates the formulation is up to standard, additional risk analysis is advised to identify control factors unrelated to the formulation, such as specialized packaging. If the formulation fails to meet either criterion A or B, it faces a high risk of contamination. In such cases, a thorough risk analysis is necessary to prevent potential contaminants during the product’s lifespan, or the formulation should be revised with new preservatives to ensure its safety.

### 4.6. Potential Skin Benefits of the S. cerevisiae Peptides (2–10 kDa)

#### In Vitro Collagenase Inhibition Assay

To evaluate the peptide’s collagenase inhibitory activity, the Collagenase Activity Colorimetric Assay Kit (Merck KGaA, Darmstadt, Germany) was used. The kit measured collagenase activity using a synthetic peptide (FALGPA) that mimics collagen’s structure. The assay was performed according to the manufacturer’s instructions. Briefly, aliquots of the peptides were prepared in the solvent, ethanol 8% (*v*/*v*) at a final concentration ranging from 50 to 1000 µg/mL. The peptides (2 µL aliquots) were then mixed with 10 µL of collagenase (0.35 U/mL), and 88 µL of assay buffer. An enzyme control containing only the enzyme and the buffer and 2 µL of solvent (ethanol 8% (*v*/*v*)) was also prepared. A collagenase inhibitor (positive control) provided by the manufacturer was used, i.e., 1,10 phenanthroline. Additionally, a blank containing only the buffer was also prepared. The reaction was started by adding 40 µL of FALGPA and 60 µL of buffer, the absorbance was then measured at 345 nm every 2 min for at least 40 min to plot a kinetic curve. Readings were performed using a BioTek SYNERGY Microplate Reader (Agilent Technologies, Santa Clara, CA, USA). Collagenase activity (enzyme control) was calculated by the following equation:

$$\mathrm{Collagenase}\;\mathrm{activity}\;(U/\mathrm{mL})=\frac{(\frac{-∆\;A345nm}{∆T}enzyme\;control-\frac{-∆\;A345nm}{∆T}\;blank)\times RV\times DF}{EC\times V}$$

∆ A345nm corresponds to the absorbance difference between the beginning and the end of the acquisition readings, ∆T corresponds to the time difference between the beginning and the end of the acquisition readings, RV is the reaction volume (0.2 mL); DF is the dilution factor; EC corresponds to the extinction coefficient of collagenase substrate (0.53 mM); V is the enzyme volume (mL).

To determine the inhibitor, i.e., 1,10-Phenanthroline or the peptides activity, the same equation to calculate the collagenase activity was applied.

The percent relative inhibition of 1,10-Phenanthroline or peptides with respect to enzyme control was calculated using this equation:

% Inhibition= [(Activity (enzyme control) − Activity (inhibitor))/Activity (enzyme control)] × 100

### 4.7. Cytotoxicity of Peptides (2–10 kDa) in Dermal Cells

The cytotoxicity of the peptides under study in dermal cells was evaluated in the melanoma cell line (MNT-1, CRL-3450, ATCC, Manassas, VA, USA), primary dermal fibroblasts (PCS-201-012, ATCC), primary epidermal keratinocytes (PCS-200-010, ATCC) and primary epidermal melanocytes (PCS-200-012, ATCC). Cultures were maintained and experiments were performed according to the cell culture. For MNT-1 and fibroblasts, it was used Dulbecco’s modified eagle medium (DMEM, ThermoFisher Scientific, Waltham, MA, USA) supplemented with a mixture of 100 µg/mL Streptomycin and 100 U/mL Penicillin (ThermoFisher Scientific) and 10% (*v*/*v*) fetal bovine serum (FBS, ThermoFisher Scientific). The medium for melanocytes and keratinocytes was Dermal cells basal media (PCS-200-030, ATCC) supplemented with melanocytes growth kit (PCS-200-041, ATCC) or keratinocytes growth kit (PCS-200-040, ATCC), respectively. In a 96-well plate, cells were seeded at a density of 7500 cells per well, and after 24 h, either crescent concentrations of the peptides or equivalent crescent concentrations of ethanol (vector control) were applied.

After a 48-h incubation at 37 °C, with 5% CO_2_ and saturated humidity, the medium was replaced with a mixture of fresh medium and the Cell Titer 96^®^ Aqueous One solution cell proliferation assay (Promega, Madison, WI, USA), following the manufacturer’s guidelines. The viability of cells exposed to each peptide concentration was then compared to the viability of cells exposed to the corresponding concentration of the vector (ethanol), with the results standardized as a percentage.

### 4.8. Wound-Healing Assay

For the wound healing assay, 4 × 10^5^ cells of primary human fibroblasts were seeded in 24 well plates in DMEM supplemented with 10% (*v*/*v*) FBS and antibiotic mixture and incubated at 37 °C, 5% (*v*/*v*) CO_2_ and 99% (*v*/*v*) humidity. Primary umbilical vein endothelial cells (HUVEC, CRL-1730, ATCC) were seeded in 24 well plates with a cell density of 4 × 10^5^ cells/mL in F12-K medium supplemented with 10% (*v*/*v*) FBS (ATCC), 1000 µg/mL Heparin (Sigma Aldrich, Merck, Darmstadt, Germany), 30 µg/mL endothelial cell growth factor (ECGF, Sigma Aldrich, Merck) and 1% (*v*/*v*) of antibiotic/antimycotic mixture and incubated at 37 °C, 5% (*v*/*v*) CO_2_ and 99% (*v*/*v*) humidity. Human dermal keratinocytes were seeded with a 2 × 10^5^ cell/mL density in 24-well plates in dermal cells basal medium (ATCC) supplemented with keratinocytes growth kit (ATCC). After 24 h, a pipette tip was used to perform a scratch in the cell monolayer, and the medium was replaced by fresh medium supplemented with 250 µg/mL of the peptides or 1% (*v*/*v*) of ethanol (vector control). The scratch was imaged for 24 h with a Cytosmart Lux2 (Axion Biosystems, Atlanta, GA, USA) and the percentage of remission was calculated by measuring the size of the wound scratch at 0 h and after 24 h with ImageJ software.

### 4.9. Inflammation Assay

To analyze the peptides (2–10 kDa) effect in the inflammation mediated by fibroblasts, normal dermal fibroblasts were seeded in T25 flasks with a cell density of 4 × 10^5^ cells/flask and after 24 h, they were incubated for 2 h with 250 µg/mL of the peptides, 1% (*v*/*v*) ethanol (vector control), or the same volume of medium (untreated control) and then for 2 h with 7 µg/mL lipopolysaccharide (LPS, Merck). Samples without LPS were prepared in parallel for control purposes. The following procedures were similar to those described in Branco et al. (2023) [15] and the expression of TNF-α in samples was determined with the 2-Ct method [84] using 18S mRNA as internal control and respective untreated control samples.

### 4.10. Endothelial Cell Tube Formation

The procedure to evaluate the in vitro angiogenesis followed the guidelines of the protocol published by Arnaoutova and Kleinman (2010) [45]. In the first step, the wells from a 96-well culture plate were coated with 50 µL Matrigel basement membrane high concentration (Corning, Merck, Corning, NY, USA) and incubated for 30 min at 37 °C. Next, HUVEC at 80% confluence in a T25 flask were detached with Tryple express (ThermoFisher Scientific) and a mixture was prepared with 1.5 × 10^5^ cells/mL and 250 µg/mL of peptides or 1% (*v*/*v*) ethanol, or prepared in F12-K medium supplemented with 5% (*v*/*v*) FBS (ATCC), 0.5 mg/mL Heparin (Sigma Aldrich, Merck), 15 µg/mL endothelial cell growth factor (ECGF, Sigma Aldrich, Merck) and 0.5% (*v*/*v*) of antibiotic/antimycotic mixture (ThermoFisher Scientific). The mixture was gently poured on top of the matrix and images were acquired each 15 min for 24 h using Cytosmart Lux2 microscope and respective software (Axion Biosystems, Atlanta, GA, USA).

### 4.11. Superoxide Anion Radical Scavenging Assay

The Superoxide anion radical scavenging activity was determined spectrophotometrically at 560 nm by monitoring the effect of the peptides (2–10 kDa) on the reduction in NBT to the blue chromogen formazan by superoxide anion, generated by the NADH/PMS system according to the procedure described by Valentão et al. (2001) [85]. Briefly, the reaction mixtures were prepared in 96 well microplates and consisted of 20 µL of solutions with different concentrations of the peptides (0 to 3.9 μg/μL), 30 μL of NADH (Alfa Aesar, Karlsruhe, Germany) (1.66 mM in phosphate buffer 19 mM, pH 7.4), 30 μL of NBT (Sigma-Aldrich, St. Louis, MO, USA) (430 μM in phosphate buffer 19 mM, pH 7.4) and phosphate buffer (19 mM, pH 7.4) up to 295 μL. The reaction was initiated by the addition of 5 μL of PMS (Sigma-Aldrich, St. Louis, MO, USA) (162 μM in phosphate buffer 19 mM, pH 7.4) and the extent of NBT reduction was followed by measuring the increase in the absorbance at 560 nm, for 2 min, at room temperature, in a microplate reader in kinetic mode (FLUOstar^®^ Omega Plate Reader, BMG Labtech, Ortenberg, Germany). Gallic acid (Alfa Aesar, Karlsruhe, Germany) was used as a positive control. Each sample (peptides or gallic acid) concentration generated a time-dependent curve and the slope (ΔAbs/sec) of the linear domain of these curves was used to determine the inhibition, in percentage, of the NBT reduction to blue chromogen formazan, according to the formula:

Inhibition (%) = [(Slope control − Slope sample)/(Slope control)] × 100

Values were assessed in triplicate and the results were expressed as the final concentration (μg/mL) in the reaction mixture, which inhibited 50% of the NBT reduction (IC_50_).

### 4.12. Statistical Analysis

Data are expressed as the average and standard deviation of at least three independent experiments. Statistical analysis was performed using GraphPad Prism v8.2.1, and data were considered statistically significant when the *p*-value < 0.05, calculated by the unpaired *t*-test.

## 5. Conclusions

In conclusion, this study underscores the multifaceted potential of the peptides secreted by *S. cerevisiae* Ethanol-red, as a promising bioactive ingredient for skincare applications. The antimicrobial activity of these peptides against a range of dermal pathogens, including *S. aureus* and *P. aeruginosa*, highlights their potential as a natural preservative. Their efficacy against Methicillin-resistant *S. aureus* (MRSA) and other pathogens such as *E. coli* and *S. epidermidis* suggests a broad-spectrum of antimicrobial capability. Moreover, the challenge test results demonstrate the efficacy of these peptides, particularly when combined with Microcare^®^ BNA at 0.3% (*v*/*v*), showing promising preservation efficacy and potentially allowing for reduced concentrations of synthetic preservatives. These findings highlight a pathway towards safer, more effective cosmetic products with optimized preservative systems, aligning with the growing consumer demand for natural and less harmful ingredients in skincare formulations.

In addition, the collagenase inhibitory properties of the peptides studied highlight their utility in anti-aging formulations, while their anti-inflammatory effects and suitability for the remodeling phase of wound healing suggest therapeutic benefits.

The capacity of the peptides (2–10 kDa) to scavenge superoxide radicals and reduce oxidative stress further supports their role in protecting the skin from oxidative damage and slowing the aging process. Taken together, these findings advocate for the continued exploration of peptides secreted by *S. cerevisiae* in skincare, leveraging their natural bioactivity to meet consumer demand for safer and more effective cosmetic products.

## Figures and Tables

**Table 1:** Minimum inhibitory concentration (MIC) of peptides (2–10 kDa) and controls against several dermal pathogens.

Strains Tested	Minimum Inhibitory Concentration (MIC) (µg/mL)				
	Peptides (2–10 kDa)	Ethanol *	Vancomycin **	Norfloxac **	Ketaconazol **
*Staphylococcus aureus*	125	n.d	1.56	-	-
Methicillin-resistant *S. aureus*	500	n.d	1.56	-	-
*Staphylococcus epidermidis*	250	n.d	1.56	-	-
*Enterococcus faecalis*	500	n.d	3.12	-	-
*Streptococcus pyogenes*	1000	n.d	3.12	-	-
*Streptococcus mitis*	500	n.d	3.12	-	-
*Bacillus cereus*	250	n.d	0.78	-	-
*Candida albicans*	1000	n.d	-	-	0.039
*Escherichia coli*	250	n.d	-	0.48	-
*Pseudomonas* *aeruginosa*	250	n.d	-	1.90	-
*Aspergillus brasiliensis*	>1000	n.d	-	-	-

* Negative control; ** Positive control; - not tested; n.d not detected.

**Table 2:** Superoxide anion radical scavenging capacity. Values are expressed as mean standard deviation.

Samples	IC_50_ (µg/mL)
Peptides (2–10 kDa)	30.6 ± 1.4
Gallic acid (positive control)	20.7 ± 3.7

## Data Availability

All data generated and analyzed in this study is included in this manuscript.

## Supplementary Materials

The following supporting information can be downloaded at: https://www.mdpi.com/article/10.3390/antibiotics13090881/s1, Movie S1. HUVEC tube formation in the presence of 250 μg/mL Peptide fraction 2–10 KDa.mp4; Movie S2. HUVEC tube formation in the presence of 1% (*v*/*v*) ethanol.mp4. Figure S1. Gel filtration chromatography profile of the 2–10 kDa peptide fraction extracted from the 7-day-old supernatant of *S. cerevisiae* Ethanol-red. Table S1. Quality control of the base formulation at t = 0 and t = 28 days for organoleptic, physico-chemical, and microbiological analysis. References [43,86] are cited in the Supplementary Materials.
