# Supplementary material for: Evaluation of Biotechnological Active Peptides Secreted by Saccharomyces cerevisiae with Potential Skin Benefits
Source: Antibiotics (Basel). 2024 Sep 13;13(9):881. doi: 10.3390/antibiotics13090881 (PMC11429205; doi:10.3390/antibiotics13090881)
Supplement: Supplementary file 1 [file antibiotics-13-00881-s001.zip › antibiotics-3161292-supplementary.pdf]

Supplementary Material

Evaluation of Biotechnological Active Peptides Secreted by *Saccharomyces cerevisiae* with Potential Skin Benefits

Elisabete Muchagato Maurício [1,2,3,*], Patrícia Branco [1,4,5,*], Ana Luiza Barros Araújo [1], Catarina Roma-Rodrigues [6,7], Katelene Lima [8], Maria Paula Duarte [9], Alexandra R. Fernandes [6,7,*] and Helena Albergaria [5]

1 BIORG—Bioengineering and Sustainability Research Group, Faculdade de Engenharia, Universidade Lusófona, Av. Campo Grande 376, 1749-024 Lisbon, Portugal
2 CBIOS—Research Center for Biosciences & Health Technologies, Universidade Lusófona, Campo Grande 376, 1749-024 Lisbon, Portugal
3 Elisa Câmara, Lda, Dermocosmética, Centro Empresarial de Talaíde, n°7 e 8, 2785-723 Lisbon, Portugal
4 Linking Landscape, Environment, Agriculture and Food (LEAF), Associated Laboratory TERRA, Instituto Superior de Agronomia, University of Lisbon, Tapada da Ajuda, 1349-017 Lisbon, Portugal
5 Unit of Bioenergy and Biorefinary, Laboratório Nacional de Energia e Geologia (LNEG), Estrada do Paço do Lumiar, 22, 1649-038 Lisbon, Portugal
6 UCIBIO—Applied Molecular Biosciences Unit, Department Ciências da Vida, NOVA School of Science and Technology, 2829-516 Caparica, Portugal
7 i4HB, Associate Laboratory—Institute for Health and Bioeconomy, Faculdade de Ciências e Tecnologia, Universidade NOVA de Lisboa, 2829-516 Caparica, Portugal
8 Research Institute for Medicines (iMed.ULisboa), Faculty of Pharmacy, Universidade de Lisboa, 1649-003 Lisbon, Portugal

9 The Mechanical Engineering and Resource Sustainability Center (MEtRICs), Chemistry Department, NOVA School of Science and Technology, Universidade NOVA de Lisboa, 2829-516 Caparica, Portugal

* Correspondence: elisabete.maurício@ulusofona.pt (E.M.M.); patricia.branco@ulusofona.pt (P.B.); ma.fernandes@fct.unl.pt (A.R.F.)

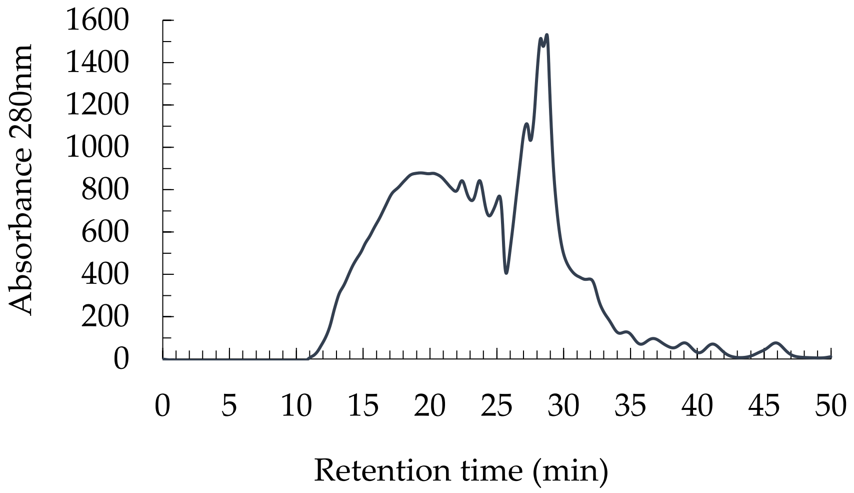

Figure S1- Gel filtration chromatography profile of the 2-10 kDa peptide fraction extracted from the 7-day-old supernatant of *S. cerevisiae* Ethanol Red

Table S1–Quality control of the base formulation at t=0 and t=28 days for organoleptic, physicochemical and microbiological analysis.

Formulation Standards	Organoleptic			Physicochemical			Microbiological					
	**Colour**	**Odour**	**General appearance**	**pH**	**viscosity**	**Physical stability**	**Mesophilic microorganisms**	***E. coli***	***S. aureus***	***P. aeruginosa***	***C. albicans***	**Yeast and moulds**
	**White**	**Perfumed**	**Creamy**	**5-6**	**18 000- 18 500 cps**	**Centrifugation 1 h at 5000 rpm**	**‹1000 CFU/g**	**n.d**	**n.d**	**n.d**	**n.d**	**‹10 CFU/g**
t=0 days	White	Perfumed	Creamy	5.2	18 000	Stable	n.d	n.d	n.d	n.d	n.d	n.d
t=28 days	White	Perfumed	Creamy	5.2	18 000	Stable	n.d	n.d	n.d	n.d	n.d	n.d

n.d - not detected; * Formulation Standards complied the SCCS guidelines [1, 2].

References

1. Huang, J.; Hitchins, A.D.; Tran, T.T.; McCarron, J.E. Microbiological methods for cosmetics. In *Bacteriological Analytical Manual (BAM)*, 8th ed.; FDA: Silver Spring, MD, USA, 2017. [**Google Scholar**]

2. Halla, N., Fernandes, I. P., Heleno, S. A., Costa, P., Boucherit-Otmani, Z., Boucherit, K., ... & Barreiro, M. F. (2018). Cosmetics preservation: a review on present strategies. *Molecules*, *23*(7), 1571.
